# Mucosal-Associated Invariant T Cell Levels Are Reduced in the Peripheral Blood and Lungs of Children With Active Pulmonary Tuberculosis

**DOI:** 10.3389/fimmu.2019.00206

**Published:** 2019-02-14

**Authors:** Clara Malka-Ruimy, Ghada Ben Youssef, Marion Lambert, Marie Tourret, Liana Ghazarian, Albert Faye, Sophie Caillat-Zucman, Véronique Houdouin

**Affiliations:** ^1^INSERM UMR1149, Centre de Recherche sur l'Inflammation, Université Paris Diderot, Paris, France; ^2^Service de Pédiatrie Générale, Hôpital Robert Debré, Assistance Publique Hôpitaux de Paris, Université Paris Diderot, Paris, France; ^3^Laboratoire d'Immunologie, Hôpital Saint Louis, Assistance Publique Hôpitaux de Paris, Paris, France; ^4^Service des Maladies Digestives et Respiratoires de l'Enfant, Hôpital Robert Debré, Assistance Publique Hôpitaux de Paris, Paris, France

**Keywords:** tuberculosis, mucosal associated invariant T cells, innate immune response, microbial infection, host pathogen interactions

## Abstract

Mucosal associated invariant T (MAIT) cells are unconventional, semi-invariant T lymphocytes that recognize microbial-derived vitamin B2 (riboflavin) biosynthesis precursor derivatives presented by the monomorphic MHC class 1-related (MR1) molecule. Upon microbial infection, MAIT cells rapidly produce cytokines and cytotoxic effectors, and are thus important players in anti-microbial defense. MAIT cells are protective in experimental models of infection and are decreased in the blood of adult patients with bacterial infections, including *Mycobacterium tuberculosis* (*Mtb*). In children, the risk of rapid progression to active tuberculosis (TB) following *Mtb* infection is higher than in adults. Whether MAIT cells influence the outcome of *Mtb* infection in children is therefore, an important issue. We analyzed MAIT cell numbers and phenotype in 115 children investigated for pulmonary TB and determined their potential correlation with disease progression. MAIT cells were reduced in numbers and activated in the peripheral blood of children with active TB as compared to those with latent TB infection (LTBI) and healthy children. Moreover, MAIT cells did not accumulate and did not proliferate in the lung of children with active TB. These results suggest that MAIT cells may be important in preventing progression of *Mtb* infection to active TB in children.

## Introduction

Tuberculosis (TB) in children remains a major diagnosis challenge, due to the non-specific nature of most clinical symptoms and lack of accurate and rapid microbiological confirmation of active *Mycobacterium tuberculosis* (*Mtb*) infection (1). Moreover, although the risk of rapid progression to active TB is higher in children than in adults, it remains very difficult to differentiate children at risk of developing active TB from those who will remain healthy and develop a latent TB infection (LTBI). Early immune responses in the lung are considered to be crucial for the outcome of *Mtb* infection.

Mucosal associated invariant T (MAIT) cells are unconventional innate-like T lymphocytes expressing a semi-invariant TCR [Vα7.2-Jα33/20/12 in humans, paired with a limited number of Vβ chains (2, 3)]. The MAIT TCR recognizes the monomorphic MHC class I-related molecule, MR1 (4), which binds riboflavin (vitamin B2) biosynthesis precursor derivatives, such as 5-OP-RU [5- (2-oxopropylideneamino)-6-D-ribitylaminouracil] produced by most bacteria and yeasts (5, 6). Upon microbial infection, MAIT cells rapidly produce inflammatory cytokines (IFNγ, TNF-α, IL-17) and mediate perforin-dependent killing of infected cells, thus participating in antimicrobial defense (7–11). Human MAIT cells can also be activated in a TCR-MR1 independent fashion in response to cytokines such as IL-12 and IL-18 (12). Cytokines produced by MAIT cells may not only act directly on infected target cells, but also promote activation of other immune cells and orchestrate adaptive immunity through dendritic cell maturation. In humans, MAIT cells are abundant in the peripheral blood, liver and mucosal tissues (lung and gut) (7, 8, 13). Their frequencies are decreased in the blood of patients with various bacterial infections (14–16), suggesting that they are recruited from the peripheral blood to the infected tissues.

Our knowledge of the role of MAIT cells in *Mtb* infection has grown in recent years (16, 17). *In vitro*, MAIT cells are activated and produce IFNγ in the presence of *Mtb*, and can kill infected epithelial cells (7–9, 18). MAIT cells are also able to inhibit BCG growth in infected macrophages (19, 20), suggesting that they may control microbial burden *in vivo*. In mice, MAIT cells promote mycobacterial clearance and participate to the early control of mycobacterial infection in the lung (7, 19). MAIT frequencies are lower in the blood of adult patients with active TB than in LTBI patients and controls (7, 8, 21, 22), suggesting that they may be a useful marker to discriminate latent from active TB. Moreover, MAIT cells are found in the lungs or pleural effusions of patients with active TB (7, 23), but whether their reduction in the periphery is related to their migration to *Mtb*-infected tissues remains unclear.

So far, studies in *Mtb*-infected children are lacking. Here, we analyzed MAIT cells numbers and phenotype in a cohort of 115 children investigated for pulmonary TB, and determined their potential correlation with the outcome of *Mtb* infection.

## Patients and Methods

### Subjects

We studied 115 consecutive HIV-negative children (6 months−16 years old) investigated for pulmonary TB because of signs suggestive of disease or recent exposure to an acute TB index case. Residual samples from systematic blood count tests were obtained at the baseline visit. The children were subsequently classified by the pediatricians based on combination of clinical, radiological and laboratory findings according to the NICE guidelines (24), as active pulmonary TB (n = 26, mean age ± sd 10.8 ± 5.1 years), infected without disease (*n* = 22, 4.9 ± 4.6 years), and exposed but non-infected (*n* = 67, 3.5 ± 3 years). Patients who had received more than 1 wk of anti-TB therapy at time of analysis were excluded. Bronchoalveolar lavage (BAL) was performed within 1 week after baseline visit in 10 active TB children suspected of airway compression or multi-resistant infection. The control population consisted of 58 healthy children (7.6 ± 5.0 years) sampled at time of pre-operative assessment.

### Flow Cytometry

MAIT cells were analyzed on 100 μl residual whole blood or BAL cell pellets within 24 h after collection, using multiparametric 10-color flow cytometry. Flow cytometry was performed using combinations of the following antibodies for 15 min at room temperature: anti-CD45 Krome Orange (clone J33, dilution 1:20), anti-CD3 ECD (UCHT1, 1:20), anti-CD8β PE or ECD (2ST8.5H7, 1:20) (all from Beckman Coulter); anti-TCR Vα7.2 FITC or APC (3C10, 1:20); anti-CD69 PeCy7 (FN50, 1:20), anti-CXCR6 PeCy7 (K041E5, 1:20), anti-CD4 AF700 (OKT4, 1:100), anti-CCR6 BV421 (G034E3, 1:20) (all from Biolegend); anti-CD161 APC (191B8, 1:20), anti-CD3 APC Vio770 (BW264156, 1:20) (all from Miltenyi); anti-CD4 APC AF750 (S3.5, 1:20, Invitrogen). For intracellular staining, cells were first stained with antibodies against surface antigens, then fixed/permeabilized, washed and incubated with anti-Ki67 PE (B56, 1:5) (BD Biosciences) at 4°C for 20 min. Data were acquired on a Navios flow cytometer (Beckman Coulter) collecting a total of at least 100,000 events in a live gate. Gates were defined through isotype and fluorescence minus one (FMO) stains. The gating strategy was CD45 vs. side scatter, and MAIT cells were defined as CD3^+^ CD4^−^ CD161^high^ Vα7.2^+^ T cells. Frequencies were expressed as a percentage of CD3^+^ lymphocytes. Absolute numbers (per microliter) were calculated from the absolute lymphocyte count determined on the same sample with a hematology automated analyzer. Where indicated, staining with the APC-conjugated MR1:5-OP-RU or MR1:6-FP tetramer (dilution 1:1,800, NIH tetramer core facility) was performed for 45 min at room temperature before surface staining with other antibodies for 20 min at 4°C. Data were analyzed using FlowJo software.

### Statistical Analysis

Two-way ANOVA on log-transformed data was used to determine the impact of age and clinical status on MAIT cell frequencies or absolute numbers. Differences between groups were analyzed using non-parametric tests for paired (Wilcoxon) and unpaired (Mann-Whitney or Kruskall-Wallis with *post-hoc* Dunn's test for multiple comparisons between all groups) analyses. Two-sided *P* < 0.05 were considered significant. Analyses were performed using Prism software v.6 (GraphPad).

## Results

**Figure 1:**
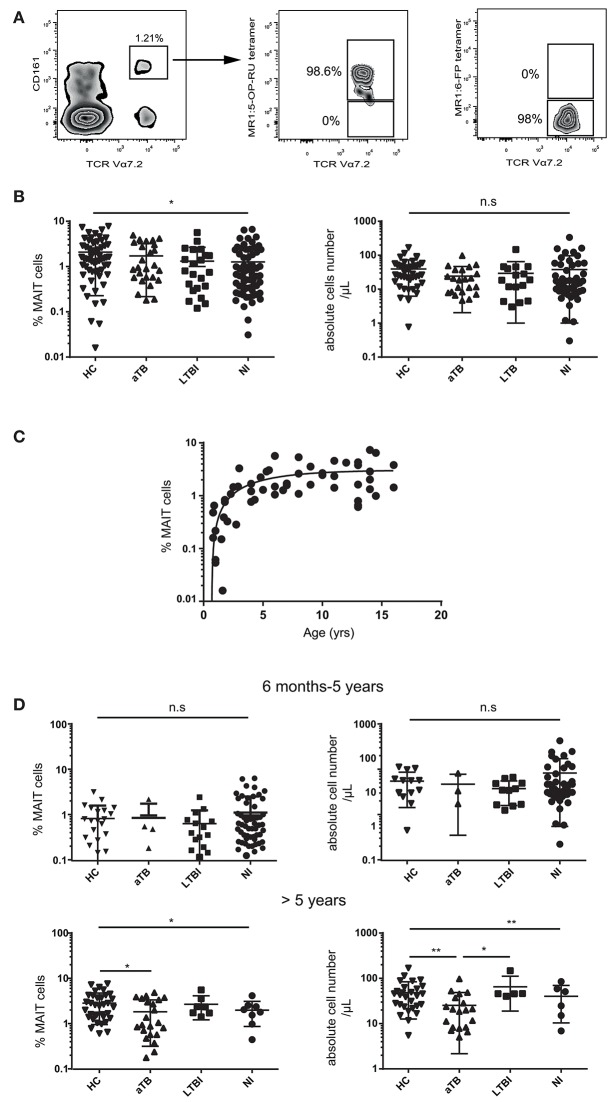
MAIT cell numbers are decreased in children with aTB over 5 years of age. **(A)** representative MAIT cell staining in a healthy child. Numbers indicate the percentage of Vα7.2^+^ CD161^high^ MAIT cells (gated on live CD3^+^ CD4^−^ T cells) (left panel) and the proportion of cells stained with the MR1:5-OP-RU tetramer (middle panel) or MR1:6-FP control tetramer (right panel). **(B)** statistical dot plots showing the percentages (left panels) and absolute numbers (right panels) of MAIT cells among CD3^+^ T lymphocytes in the whole children cohort. **(C)** relationship between log10-based transformed MAIT cell percentages and age in 58 healthy children aged 6 months to 16 years. **(D)** percentages and absolute numbers of MAIT cells in children 6 mo-5 years old (upper panel) and over 5 years (lower panels). Acute TB (aTB), latent TB infection (LTBI), exposed non-infected (NI) children, and healthy children controls (HC). Geometric means (horizontal bars) and statistical significance (Kruskall-Wallis test with *post-hoc* Dunn's test for multiple comparisons between all groups) are indicated. ^*^P < 0.05; ^**^P < 0.01; n.s, not significant.

We quantified peripheral blood MAIT cells in 115 children investigated for pulmonary TB and further classified as acute TB (aTB), LTBI, and exposed non-infected (NI) children, in comparison to 58 healthy children (HC). MAIT cells were identified by flow cytometry as CD3^+^CD4^−^Vα7.2^+^CD161^high^ T cells. This population fully overlapped with the MR1:5-OP-RU tetramer^+^ population, as we previously showed in infants more than 6 months old (25) (Figure 1A).

The proportion of MAIT cells differed among children groups (*P* = 0.011), but there was a high variability between individuals (Figure 1B). We recently demonstrated that MAIT cells (which are very few at birth) slowly expand during infancy, requiring at least 5–6 years to reach adult levels (25), an observation confirmed in the present study (Figure 1C). As the median ages for the different groups were not comparable (aTB were older than other groups, *p* < 0.0001), the observed heterogeneity of MAIT cell values could be due to age differences. Indeed, age dichotomized at 5 years was the only significant source of variation of MAIT cell values in two-way ANOVA (*P* < 0.0001). We therefore, analyzed MAIT cells separately in young (< 5 years old) and older children (>5 years). Differences were restricted to children over 5 years old, among whom those with active TB had fewer MAIT cells than HC (*P* = 0.004) and LTBI children (*P* = 0.012; Figure 1D). MAIT cell numbers were not correlated with *Mtb* culture positivity (*P* = 0.34).

**Figure 2:**
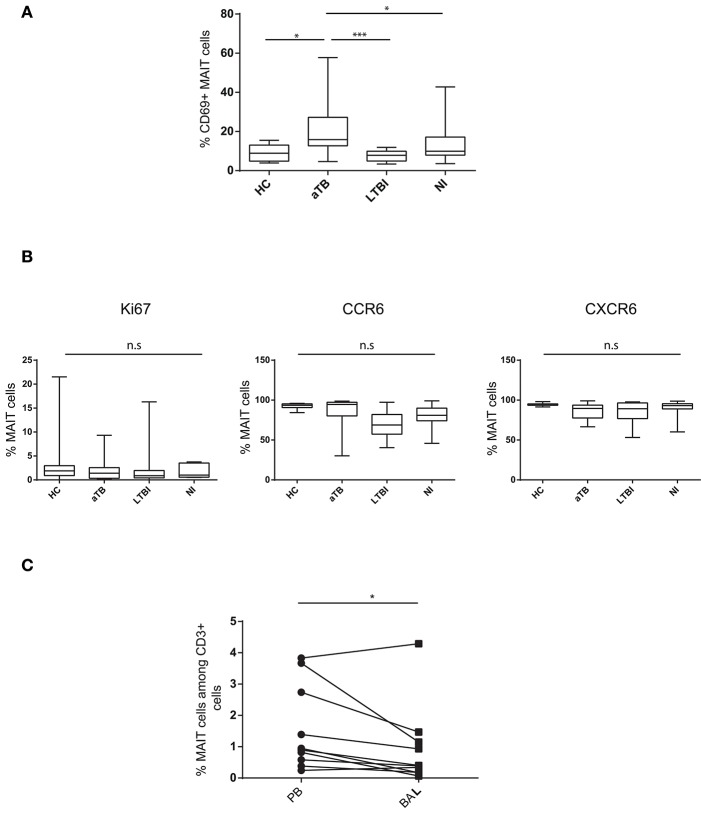
MAIT cells are activated in the peripheral blood and do not accumulate in the lung in children with active TB. **(A)** box and whisker plots show median, interquartile range and the minimal to maximal proportions of MAIT cells expressing CD69 (Mann-Whitney test). **(B)** box and whisker plots show median, interquartile range and the minimal to maximal proportions of MAIT cells expressing the indicated marker in the different children groups. Differences were not significant (Mann-Whitney test). **(C)** correlation of MAIT cell frequencies in the peripheral blood (PB) and bronchoalveolar lavage (BAL) in aTB children (Wilcoxon matched-pairs signed rank test). Each point corresponds to 1 patient and lines connect matched samples. ^*^P < 0.05; ^***^P < 0.0001, n.s, not significant.

We next determined if MAIT cells displayed particular phenotype characteristics during *Mtb* infection, by analyzing expression of activation (CD69) and proliferation (Ki67) markers, and tissue-homing (CCR6, CXCR6) chemokine receptors. In children with active TB, the frequency of CD69^+^ MAIT cells was higher than in LTBI (20.7 vs. 7.5%, *P* < 0.0001) and HC children (9.1%, *P* = 0.007) (Figure 2A). The proportion of CD69+ cells was not correlated with the frequency of circulating MAIT cells (*P* = 0.91). Very few Ki67+ were observed, and expression of chemokine receptors was comparable in the different children groups (Figure 2B). Taken together, these results indicate MAIT cells are reduced in numbers and activated in the peripheral blood of children with active TB.

MAIT cells are abundant in the lung mucosa from healthy subjects (26). To determine if reduction in peripheral blood MAIT cell levels was a consequence of their recruitment to infected lung, we quantified MAIT cells in BAL fluid from 10 children with active TB. MAIT cell frequencies were lower in BAL than in paired peripheral blood samples (0.93 and 1.55% of T cells, respectively, *p* = 0.027) (Figure 2C). In tissues, CD69 is a marker of resident memory T cells and accordingly, was expressed on the vast majority of MAIT cells from BAL. There was no increase in Ki67^+^ MAIT cells in BAL compared to peripheral blood (2.46 and 2.81%, respectively), suggesting that MAIT cells did not expand locally during active TB. Expression of homing markers was comparable in BAL and peripheral blood (not shown). Taken together, these results do not support the hypothesis that MAIT cells are recruited to and/or expand in the *Mtb*-infected bronchial epithelium in children.

## Discussion

In contrast to adults, pediatric active TB most often results from progression of a primary *Mtb* infection rather than from reactivation of a latent infection. Since MAIT cells are able to rapidly respond to microbes before the maturation of the specific memory adaptive immunity, they could be crucial for the control of *Mtb* infection in children.

Our results show that MAIT cells are numerically deficient in the peripheral blood of children with active TB as compared to LTBI and healthy children. Notably, because of age-related expansion of MAIT cell numbers and age differences in our patient groups, this observation was restricted to children over 5 years old. Whether MAIT cell number could be used as a reliable marker to discriminate latent from active TB will require longitudinal studies in larger cohorts of patients to provide convincing results.

It seems unlikely that the low number of circulating MAIT cells is related to their recruitment to the *Mtb*-infected bronchial epithelium, as there were even fewer MAIT cells in BAL than in matched peripheral blood. Moreover, MAIT cells did not appear to expand locally, as indicated by their absence of Ki67 expression. It cannot be excluded that measurements performed at the baseline visit missed a transient expansion, as observed in the blood of *Mtb*-infected macaques 3–4 weeks after intra-bronchial infection (27). Alternatively, the low number of MAIT cells in aTB children, both in the peripheral blood and BAL, may be due to activation-induced cell death upon *Mtb* infection, as suggested by the presence of activated circulating MAIT cells. Only a longitudinal analysis, not feasible in children, could characterize the kinetics of MAIT cell frequency in paired peripheral blood and BAL samples, and determine if MAIT cell redistribution is reversible upon successful treatment. The low frequency of MAIT cells in the broncho-alveolar compartment after *Mtb* infection contrasts with the very dramatic pulmonary MAIT cell expansion recently described in mice following infection with *Legionella longbeachae*, another intracellular pathogen (28). Whether MAIT cells accumulate in the lung parenchyma or granulomas in aTB patients could not be verified because lung biopsies were not available. However, a recent study in *Mtb*-infected Rhesus macaques (29) corroborates our findings, by showing little evidence of MAIT cell accumulation and activation in the airways, and the presence of very few MAIT cells within *Mtb* granulomas. It seems unlikely that a difference in the host species (mouse vs. human/macaques) can explain these discordant observations, as the MR1-MAIT cell axis is highly conserved in mammals. Rather, expansion and activation of MAIT cells in the lungs could vary depending on the concentration of microbial-derived MR1 ligands, which differs between bacterial species, as nicely demonstrated for commensal bacteria (30). *Mtb*, one of the most successful of human pathogens, has acquired the ability to establish latent or progressive infection and persist even in the presence of a fully functioning immune system. This likely reflects a highly evolved program of immune evasion strategies, which may include modulation of the level of riboflavin or the type of riboflavin metabolites (inhibitory or activating) produced by *Mtb* to prevent or alter the quality of MAIT cell responses.

Lastly, one cannot exclude that a low MAIT cell number may, by itself, promote disease progression. MAIT cell frequency may reflect the tendency of the immune system toward efficient anti-microbial T cell responses and correlate with protection from progression to active TB. Thus, a near-complete specific deficiency in circulating MAIT cells was reported in a 22-old patient who died from severely impaired control of bacterial respiratory infections (31). Of note, a MR1 gene polymorphism, associated with lower MR1 expression, was recently associated with susceptibility to meningeal tuberculosis in Vietnamese adult patients (32). It will be crucial to know if such association is observed in other populations, to determine if deficient MR1-antigen presentation to MAIT cells is involved in susceptibility to TB.

In conclusion, our results provide significant support to the role of MAIT cells in modifying the clinical phenotype of microbial infections, in particular in preventing the progression of active TB from *Mtb* infection in children. Unraveling the mechanisms by which *Mtb* evades or modulates MAIT cell activation could be of critical importance for the development of new vaccines based on live attenuated mycobacterial strains.

## Ethics Statement

The study was approved by the institutional review board (CEERB-RD N°2014/121). All subjects (or their parents) provided written informed consent.

## Author Contributions

CM-R, GB, ML, MT, and LG conducted experiments and analyzed data. AF and VH provided patient samples. SC-Z and VH designed research studies, supervised the study, analyzed data, and wrote the manuscript with the help of other coauthors. CM-R and GB contributed equally to the present study.

### Conflict of Interest Statement

The authors declare that the research was conducted in the absence of any commercial or financial relationships that could be construed as a potential conflict of interest.
